# Physical and Functional Interaction of Mitochondrial Single-Stranded DNA-Binding Protein and the Catalytic Subunit of DNA Polymerase Gamma

**DOI:** 10.3389/fgene.2021.721864

**Published:** 2021-09-01

**Authors:** Grzegorz L. Ciesielski, Shalom Kim, Carolina de Bovi Pontes, Laurie S. Kaguni

**Affiliations:** ^1^Department of Biochemistry and Molecular Biology and Center for Mitochondrial Science and Medicine, Michigan State University, East Lansing, MI, United States; ^2^Institute of Biosciences and Medical Technology, University of Tampere, Tampere, Finland; ^3^Department of Chemistry, Auburn University at Montgomery, Montgomery, AL, United States

**Keywords:** mitochondrial DNA replication, DNA polymerase gamma, mitochondrial single-stranded DNA-binding protein, mitochondrial biogenesis, intermolecular interactions

## Abstract

The maintenance of the mitochondrial genome depends on a suite of nucleus-encoded proteins, among which the catalytic subunit of the mitochondrial replicative DNA polymerase, Pol γα, plays a pivotal role. Mutations in the Pol γα-encoding gene, *POLG*, are a major cause of human mitochondrial disorders. Here we present a study of direct and functional interactions of Pol γα with the mitochondrial single-stranded DNA-binding protein (mtSSB). mtSSB coordinates the activity of the enzymes at the DNA replication fork. However, the mechanism of this functional relationship is elusive, and no direct interactions between the replicative factors have been identified to date. This contrasts strikingly with the extensive interactomes of SSB proteins identified in other homologous replication systems. Here we show for the first time that mtSSB binds Pol γα directly, in a DNA-independent manner. This interaction is strengthened in the absence of the loop 2.3 structure in mtSSB, and is abolished upon preincubation with Pol γβ. Together, our findings suggest that the interaction between mtSSB and polymerase gamma holoenzyme (Pol γ) involves a balance between attractive and repulsive affinities, which have distinct effects on DNA synthesis and exonucleolysis.

## Introduction

Replication of the mitochondrial genome depends on a set of nucleus-encoded proteins (Ciesielski et al., 2016b). The synthesis of mitochondrial (mt)DNA is catalyzed by the DNA polymerase gamma holoenzyme (Pol γ), which in vertebrates comprises the catalytic subunit, Pol γα, and a dimeric accessory subunit, Pol γβ_2_ (Kaguni, 2004; Ciesielski et al., 2016b). Notably, mutations in the nuclear *POLG* gene encoding Pol γα are the most common cause of human mitochondrial diseases identified to date (Stumpf et al., 2013). We, and others, have demonstrated previously that the activity of Pol γ is facilitated by mtSSB (Gray and Wong, 1992; Korhonen et al., 2004; Oliveira and Kaguni, 2011), which ensures optimal organization of the single-stranded (ss)DNA template (Ciesielski et al., 2015), and a maximal synthetic rate by Pol γ (Cerrón et al., 2019).

The structural organization of mtSSB resembles that of bacterial SSB proteins, and it exhibits the same DNA binding modality (Morin et al., 2017; Oliveira and Ciesielski, 2021). However, whereas bacterial homologues have been demonstrated to play a direct role in the recruitment of the genome replication factors, no such physical interactions of mtSSB have been documented (Oliveira and Ciesielski, 2021). This appears to correspond with the lack of a large C-terminal domain in mtSSB, which in the homologous SSB of *Escherichia coli*, as well as in cases of viral SSB proteins (e.g., phages T7 and T4), mediates the binding of replication factors (Curth et al., 1996; Raghunathan et al., 2000; Salinas and Benkovic, 2000; Hernandez and Richardson, 2019; Oliveira and Ciesielski, 2021). On the other hand, a recent real-time kinetic analysis suggested that a strong, specific interaction of mtSSB with Pol γ is needed to dislodge the former from the DNA template, and ensure the maximal rate of DNA synthesis. In the absence of the putative interaction, the DNA synthesis rate of Pol γ is significantly reduced (Cerrón et al., 2019). These results suggest that mtSSB may interact with replication factors directly, despite the lack of the C-terminus.

**Figure 1:**
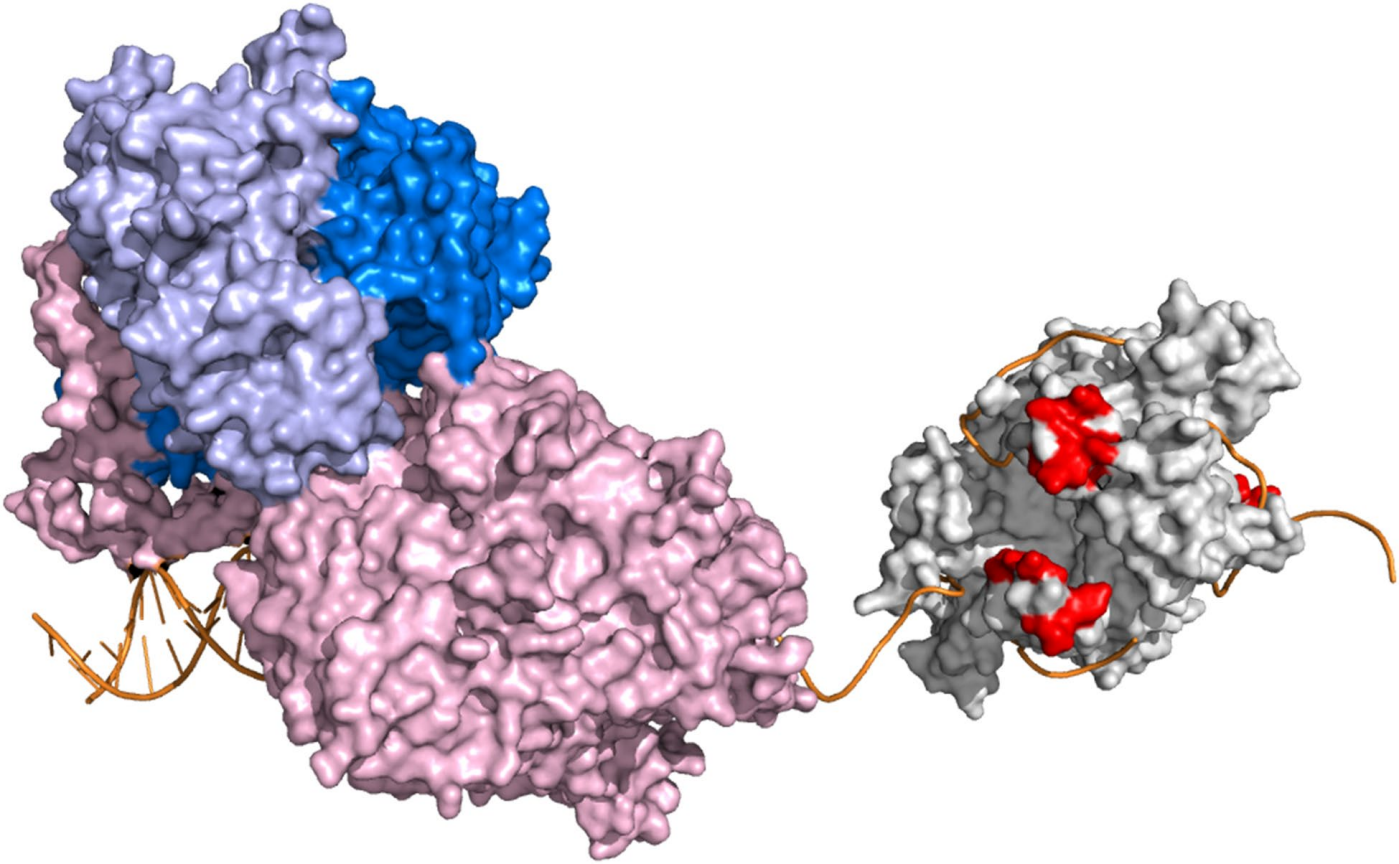
Conceptual representation of DNA synthesis by Pol γ on a mtSSB-bound ssDNA template. Pol γ holoenzyme and mtSSB tetramer are represented by crystal structures PDB:4ZTZ (Szymanski et al., 2015) and PDB:6RUP (Piro-Mégy et al., 2019), respectively. The catalytic subunit, Pol γα, is represented in light pink. The dimeric accessory subunit, Pol γβ_2_, is depicted in two shades of blue. The Pol γ-bound primer-template is oriented as in the original crystal structure. The mtSSB tetramer is represented in light grey. The acidic residues of the loop 2.3 structure of mtSSB (S67, D69, S70, E71, Y73, Q74, and D77) are shown in red. ssDNA was modeled onto mtSSB as described previously (Oliveira and Kaguni, 2011), by aligning ssDNA chains of the *Escherichia coli* SSB crystal structure (PDB:1EYG; Raghunathan et al., 2000) with the mtSSB tetramer structure. Orientation of mtSSB is dictated by the directionality of the modeled ssDNA. The software Pymol (www.pymol.org, Schrödinger, LLC) was used to create the figure. The scheme is not meant to detail structural and/or functional aspects of the replisome components.

We have shown previously that the loop 2.3 structure of mtSSB, which is conserved among vertebrates, is necessary for the stimulation of the activity of Pol γ holoenzyme (Oliveira and Kaguni, 2011). Loop 2.3 of human mtSSB encompasses 12 amino acids, most of which are disordered in the crystal structure (Yang et al., 1997). The loop extrudes from the surface of the quaternary structure and carries a negative charge as indicated by electrostatic surface potential (Oliveira and Kaguni, 2011), resembling the character of the C-terminus of *Ec*SSB. Our earlier structural predictions suggested that loop 2.3 might engage in a physical interaction with Pol γ holoenzyme (Oliveira and Kaguni, 2011). In this study, we sought to evaluate a putative mtSSB-Pol γ interaction and its relevance for DNA synthesis (Figure 1).

## Materials and Methods

Nucleotides, nucleic acids and proteins, available in the Supplementary Materials.

### Biolayer Interferometry

**Figure 2:**
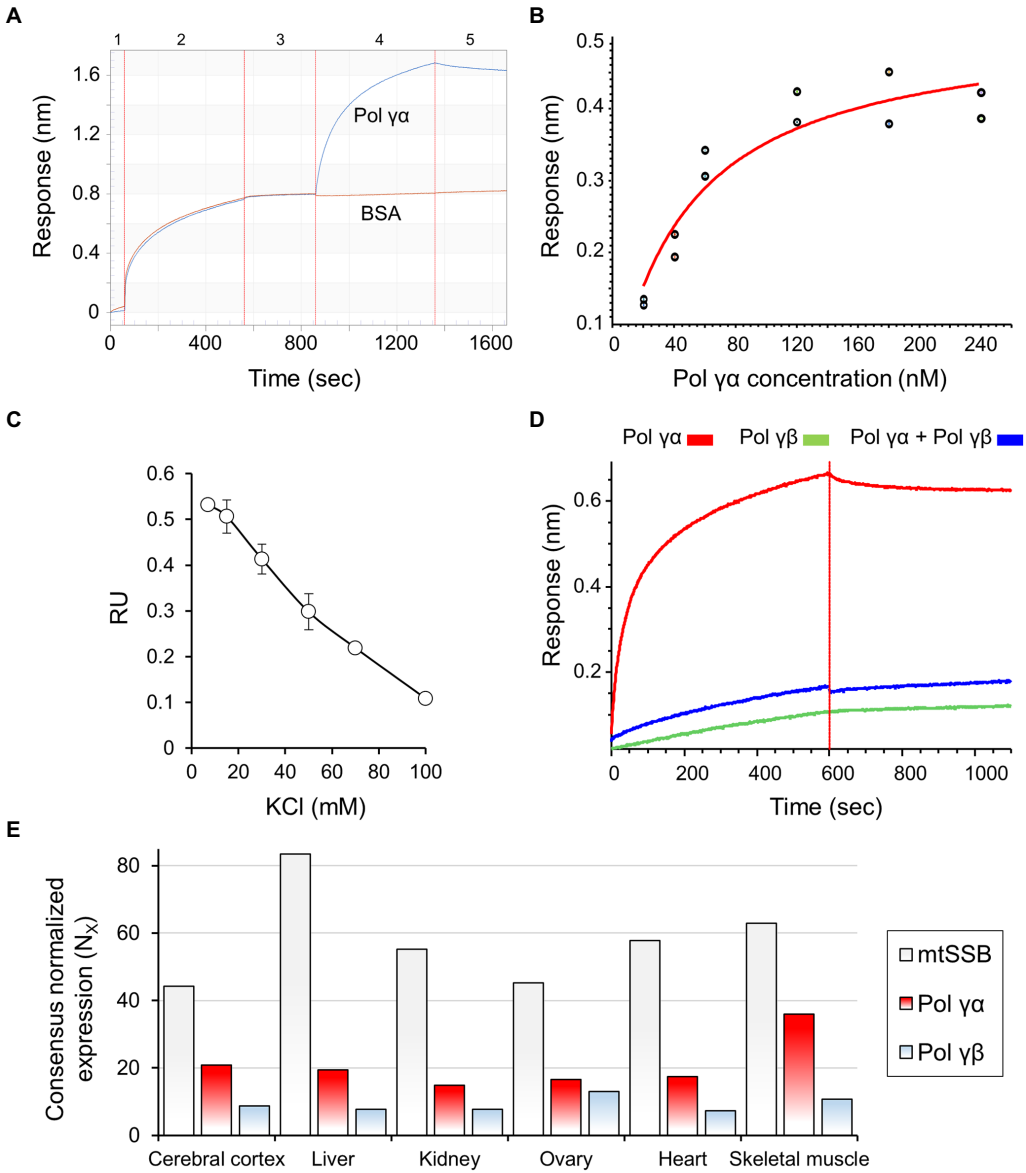
mtSSB interacts physically with Pol γα but not Pol γ holoenzyme. **(A)** The affinity of mtSSB for Pol γα was assessed by biolayer interferometry (BLI), as described under Materials and Methods. A representative sensogram is shown, and the numbers atop indicate individual experimental steps. Briefly, hydrated biosensors (1) were saturated with mtSSB (2), blocked with BSA (3), and placed into solutions of Pol γα. The association of the proteins was measured in real-time for 5min (4), after which the sensors were moved to a buffer to allow dissociation (5). Excess BSA was present in all solutions used in steps 3–5. **(B)** The binding affinity of mtSSB for Pol γα was determined by BLI, as described in **(A)**, using two sets of association responses upon increasing Pol γα concentrations (indicated), fitted to a 1:1 binding model (see also Supplementary Figure 2). **(C)** The effect of salt concentration on the stability of the Pol γα-mtSSB complex was assessed by measuring changes in the association response of 120nM Pol γα in the presence of increasing KCl concentrations. **(D)** The association of Pol γβ with mtSSB, and its effect on the mtSSB-Pol γα binding, was assessed as described in **(A)**, replacing or preincubating 120nM Pol γα with 120nM Pol γβ_2_, as indicated. **(E)** The data representing the consensus transcript expression values (N_X_) of Pol γα, Pol γβ, and mtSSB in selected tissues, were obtained from Human Protein Atlas. Pol γα corresponds to entry *POLG* (https://www.proteinatlas.org/ENSG00000140521-POLG/tissue); Pol γβ to entry *POLG2* (https://www.proteinatlas.org/ENSG00000256525-POLG2/tissue); mtSSB to *SSBP1* (https://www.proteinatlas.org/ENSG00000106028-SSBP1/tissue). The Consensus Normalized eXpression (NX) levels were created by the Human Protein Atlas team, by combining the data from the three transcriptomics datasets (HPA, GTEx, and FANTOM5) using the internal normalization pipeline.

Biolayer interferometry experiments were performed on Octet RED384 device (Fortebio). Streptavidin-sensors were hydrated in PBS for 10min and the baseline was recorded for 1min. Sensors were next saturated with a ligand: 600nM BTN-mtSSB or BTN-mtSSB_l2.3_, or 1μM BTN-ssDNA 40-oligomer (as indicated), for at least 5min. Functionalized sensors were blocked in Ac buffer: 50mM Tris–HCl pH 8.0, 10mM KCl, 4mM MgCl_2_, 0.4mg/ml BSA, 10mM DTT, for 5min. Association reactions were carried out subsequently in the Ac buffer with the addition of indicated analyte, for 10min. After that, sensors were placed back into the Ac buffer for 10min to record dissociation. All the steps were performed at 30°C and 1,000rpm shaking rate. An exemplary experiment is represented in Figure 2A. The binding and dissociation curves were fitted to a 1:1 interaction model in case of the mtSSB-Pol γα binding, and mass transfer model in case of ssDNA-mtSSB binding.

### Processive and Gap-Filling DNA Synthesis Assay

**Figure 3:**
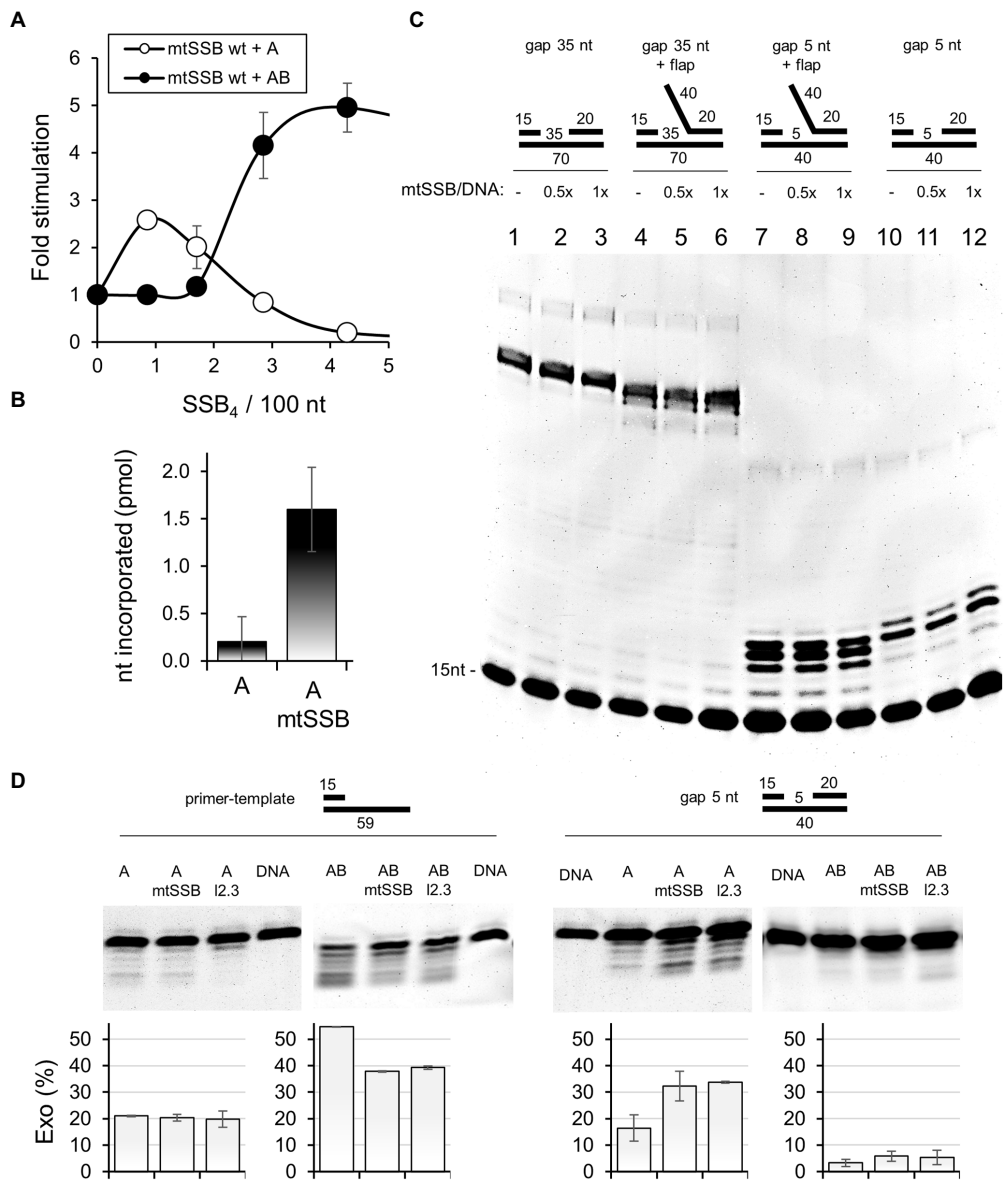
mtSSB stimulates the activity of Pol γα. **(A)** The processive DNA synthesis assay was performed as described under Materials and Methods, using 55 fmol singly-primed M13 circular DNA (6,407nt), 20 fmol of Pol γα (A, open circles) or Pol γ holoenzyme (AB, closed circles) and increasing amounts of mtSSB: 0, 3, 6, 10, and 15pmol. Assays were performed at 30mM KCl and 4mM MgCl_2_. The data represent the mean of three experiments, ±SD. The results were normalized to the amount of nucleotide incorporated by Pol γα or Pol γ holoenzyme in the absence of mtSSB (arbitrarily set to 1 in each case). **(B)** Nucleotide incorporation on DNase I-activated calf thymus DNA was assessed as described under Materials and Methods, using Pol γα (A), in the presence or absence of mtSSB, as indicated. The data represent the mean of three experiments, ±SD. **(C)** The single gap assay was performed as described under Materials and Methods in the absence or presence of 10 or 20nM mtSSB_4_, represented as their molar ratio to available binding sites on the DNA substrate (mtSSB/DNA). DNA substrates with a 5 or 35nt gap, with or without a 40nt flap were used as indicated. Products of reactions were analyzed by denaturing polyacrylamide gel electrophoresis. The gel image is representative of three independent analyses. **(D)** The exonuclease assay was performed as described under Materials and Methods, using Pol γα (A) or Pol γ holoenzyme (AB), in the presence or absence of 20nM mtSSB_4_ or mtSSB_l2.3_. DNA substrate with a 5nt gap, or primer-template (15/59) were used as indicated. Products of reactions were analyzed by denaturing polyacrylamide gel electrophoresis. The relative abundance of the products of exonucleolysis was estimated by densitometry and the mean of three independent experiments (±SD) is presented in the graphs below.

**Figure 4:**
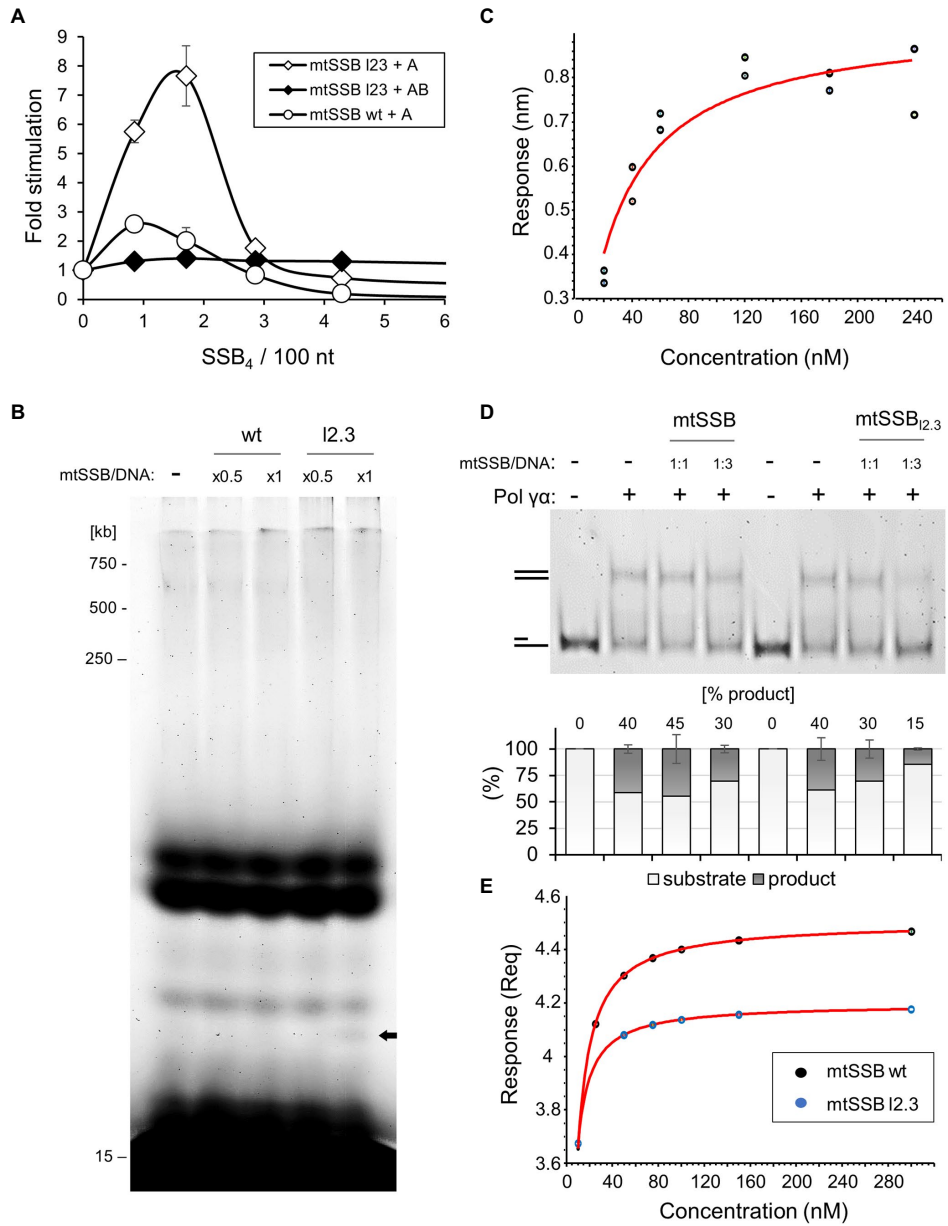
The lack of loop 2.3 impedes the ability of Pol γ to displace mtSSB. **(A)** The processive DNA synthesis assay was performed as described in Figure 3A except that the mtSSB_l2.3_ variant was used instead of the wild-type mtSSB where indicated. **(B)** The processivity assay was performed as described under Materials and Methods, using singly-primed M13 DNA, Pol γα, and increasing concentrations of the wild-type or mtSSB_l2.3_ variant, at the indicated molar ratios to available binding sites on the DNA substrate (mtSSB/DNA). Products of reactions were analyzed by denaturing polyacrylamide gel electrophoresis. **(C)** The binding affinity of mtSSB_l2.3_ for Pol γα was determined by BLI, as described in Figure 2B, using two sets of association responses upon increasing Pol γα concentrations (indicated), fitted to a 1:1 binding model. **(D)** The ability of Pol γα to displace wild-type or loop2.3-deficient mtSSB was assessed by primer extension assay on a primer-template (15/59) DNA substrate, at the indicated molar ratio of mtSSB variant to available binding sites on the DNA substrate (mtSSB/DNA). The products were analyzed by native polyacrylamide gel electrophoresis. The relative abundance of the fully double-stranded product was estimated by densitometry and the mean of three independent experiments (±SD) is presented in the graph below. **(E)** ssDNA-binding affinity of the wild-type and mtSSB_l2.3_ variant was measured by biolayer interferometry, as described in Materials and Methods. The association traces indicated a non-1:1 binding and mass transfer interference. The DNA-binding affinities were estimated, therefore, from the equilibrium responses of the mass transfer fits (Supplementary Figure 5).

The processive DNA synthesis assay (Figures 3A, 4A) was performed as described previously (Ciesielski et al., 2015). The calf-thymus DNA gap-filling assay (Figure 3B) was modified from (Oliveira and Kaguni, 2009). Twenty-five microliters reaction mixtures contained 50mM Tris-HC1 pH 8.5, 4mM MgCl_2_, 400μg/ml BSA, 10mM DTT, 30mM KCl, 30μM (each) dNTPs mix, [α-^32^P]dCTP (2μCi), 25μg/ml DNase-I activated calf thymus DNA, 1nM Pol γα, and 850nM mtSSB_4_. The assay was carried out at 30°C for 10min. Samples were processed as described previously (Oliveira and Kaguni, 2009), and nucleotide incorporation was quantified in a liquid scintillation counter.

### Processivity Assay

The processivity assay (Figure 4B) is a modification of the processive DNA-synthesis assay. Reaction mixtures of 20μl total volume contained 50mM Tris–HCl pH 8.5, 4mM MgCl_2_, 400μg/ml BSA, 10mM DTT, 30mM KCl, 1mM mix of dGTP, dTTP, dCTP, 0.5mM dATP, 1mM Cy3-dUTP, 5nM of singly-primed M13 DNA, 5nM Pol γα, and 425nM or 850nM of mtSSB_4_ variant. Assays were carried out at 37°C for 30min and analyzed on 6% denaturing (7M Urea) polyacrylamide gels.

### mtSSB Displacement Assay

The mtSSB displacement was inferred from the efficiency of primer extension (Figure 4D) carried out in a 20μl reaction mixture containing 50mM Tris HCl pH 8.5, 20mM KCl, 10mM DTT, 4mM MgCl_2_, 0.1mg/ml BSA, 10% glycerol, 100μM (each) dNTPs mix, 20nM fluorescently-labeled primer-template (15/59) DNA substrate, 5nM Pol γα, and 20nM or 60nM of mtSSB_4_ variant. Assays were performed at 37°C for 30min, and products were analyzed by native 12% polyacrylamide electrophoresis.

### Single Gap and Exonuclease Assays

The single gap and exonuclease assays were carried out under the same conditions as the mtSSB displacement assay, except that fluorescently-labeled single gap DNA substrates indicated in Figures 3C,D, were used. The single gap assay was carried out in the presence or absence of 10 or 20nM mtSSB_4_, for 10min. The exonuclease assay was performed in the absence of dNTPs in the reaction mixture, in the presence or absence of 20nM mtSSB or mtSSB_l2.3_, for 30min. Where indicated, Pol γ holoenzyme was used instead of Pol γα, at equivalent concentration. Samples were analyzed by denaturing 18% polyacrylamide gel electrophoresis.

## Results

### Direct Interaction Between mtSSB and Pol γα Is Abolished by the Pol γβ Subunit

We examined the putative physical interaction between Pol γ and mtSSB by biolayer interferometry (Ciesielski et al., 2016a), using purified recombinant proteins. To utilize mtSSB as the ligand, we conjugated it with biotin (see Materials and Methods; Supplementary Figure 1A), and documented that the modification did not affect its ability to stimulate Pol γ activity *in vitro* (Supplementary Figure 1B). Then, streptavidin-coated biosensors were saturated with biotinylated (BTN-)mtSSB and used to assess the affinity of analytes in real-time (see Materials and Methods for details). We observed that Pol γα binds to mtSSB-sensors with a dissociation constant of K_D_=48nM (±9.9nM; Figures 2A,B). The specificity of the binding can be inferred from the fact that it occurs in spite of the presence of excess bovine serum albumin (BSA), which alone exhibited no affinity for the functionalized sensors (Figure 2A). The physical mtSSB-Pol γα interaction was shown to be electrostatic in nature, as increasing salt concentration eliminates it (Figure 2C). In contrast to Pol γα, we observed that Pol γβ does not bind to mtSSB-sensors under our experimental conditions (Figure 2D). Moreover, preincubation of Pol γα with Pol γβ at a 1α: 1β_2_ molar ratio abolished the Pol γα-mtSSB binding (Figure 2D).

In the context of the mtDNA replication, functional interaction between Pol γ and mtSSB occurs at the DNA template. We tested whether the presence of ssDNA affects the observed interaction. To that end, mtSSB-sensors were incubated in solutions containing increments of 43-nt-long ssDNA oligomers, and then placed into the Pol γα solution; we observed no effect of pre-binding of ssDNA on the capacity of mtSSB to bind Pol γα (Supplementary Figures 2, 3).

### mtSSB Stimulates the Activity of Pol γα by Directing It to the Primer Site

While the cooperative function of the Pol γα and Pol γβ subunits is pivotal for mtDNA replication, comprehensive transcriptomics analysis, presented in the Human Protein Atlas^1^ (Uhlen et al., 2015, 2017; Thul et al., 2017), demonstrates that in the majority of tissues, Pol γα is expressed to a greater extent than Pol γβ (roughly ~2-fold, Figure 2E). This implies that Pol γα, and its interaction with mtSSB, may have an independent physiological role.

To assess the functional relevance of the mtSSB-Pol γα interaction, we first tested the efficiency of processive DNA synthesis by Pol γα on a singly-primed M13 ssDNA template, in the presence of increments of mtSSB (Figure 3A). We observed that at concentrations lower than that needed for DNA template saturation, mtSSB stimulates modestly DNA synthesis by Pol γα. At concentrations approximating that needed to saturate the ssDNA template, mtSSB inhibited Pol γα activity. In contrast, Pol γ holoenzyme was significantly more efficient and its activity was stimulated when levels of mtSSB exceeded that required for template saturation, in agreement with our previous report (Ciesielski et al., 2015). These observations indicate that the stimulatory effect of mtSSB on Pol γα likely results from limiting the binding of Pol γα to ssDNA, effectively directing Pol γα to the primer site. The inhibition of Pol γα at higher mtSSB concentrations suggests that, in contrast to the holoenzyme, Pol γα exhibits limited ability to displace mtSSB from the DNA template.

It has been previously suggested that Pol γα may be engaged in gap-filling during mtDNA repair (Longley et al., 1998a; Pinz and Bogenhagen, 2006; Kazak et al., 2012). In support of this, Pol γα exhibits a relatively high processivity of ~100nt (Longley et al., 1998b; Johnson et al., 2000), as compared to that of 1–15nt of other DNA polymerase catalytic subunits (McHenry and Kornberg, 1977; Hori et al., 1979; Lee et al., 2010), and exhibits higher fidelity than Pol γ holoenzyme (Longley et al., 2001). To test whether the interaction with mtSSB might facilitate the gap-filling activity of Pol γα, we tested the nucleotide incorporation rate of Pol γα on DNase I-activated calf-thymus (ct)DNA, which contains nicks, short ssDNA gaps, and an overall high primer density (Wernette et al., 1988), in the presence and absence of mtSSB (Figure 3B). We found that the presence of mtSSB resulted in an ~8-fold increase in nucleotide incorporation by Pol γα. To evaluate in detail the role of mtSSB in the gap-filling activity of Pol γα, we performed a primer-extension assay using DNA substrates containing either a 5 or 35nt gap, with or without a downstream 5'-flap (Figure 3C). The 35nt gap as well as the 40nt 5'-flap were designed to allow binding of a single mtSSB molecule, in accordance with its DNA-binding size under the experimental conditions used (i.e., at low salt; Morin et al., 2017). We observed no qualitative or quantitative differences between DNA products generated by Pol γα in the presence or absence of mtSSB on any of the DNA substrates used (Figure 3C). This indicates that the interaction of mtSSB with Pol γα does not affect the gap-filling activity directly, and the increase in activity in the ctDNA assay likely resulted from restricting the non-productive binding of Pol γα to ssDNA stretches, as in the case of the processive DNA synthesis assay. This analysis also showed that the interaction does not promote the strand-displacement DNA synthesis, as in the absence of the 5'-flap, Pol γα synthesized products only 1nt longer than the gap size, which corroborates a previous report showing that Pol γα has a modest ability to melt the DNA duplex (He et al., 2013). Here we show, however, that this ability is counteracted by the presence of the 5'-flap. In addition, we observed no apparent reduction in the quantity of the products generated over the 35nt gap in the presence of mtSSB, which suggests that Pol γα alone is able to displace a single mtSSB from the DNA template.

In addition to its polymerase activity, Pol γα is capable of exonucleolytic activity, which in addition to its role in proofreading during DNA replication, may also be relevant for excision-based mtDNA repair. Notably, excision of certain lesions, such as 8-oxoguanine, can be catalyzed by Pol γα only in the absence of Pol γβ (Wong et al., 2009). To assess whether the interaction with mtSSB may facilitate the exonucleolytic activity of Pol γα, we tested primer excision in the presence and absence of mtSSB, using a DNA substrate containing a 5nt gap (Figure 3D). We observed that mtSSB stimulated primer excision, which considering the lack of mtSSB binding sites on this DNA substrate, can be explained by a direct participation of mtSSB in Pol γα loading. To test this, we repeated the experiment in the presence of Pol γβ. We observed that the Pol γ holoenzyme was virtually incapable of excising the primer on this substrate, and the presence of mtSSB did not affect this. This indicates that an interruption of the Pol γα-mtSSB interaction by the stronger competitor impedes utilization of the gapped substrate by Pol γα, hence supporting the relevance of the interaction for loading of Pol γα onto short gap sites. In addition, we performed the exonuclease assay using a primer-template substrate (Figure 3D). Notably, in this case, the exonucleolytic activity of the holoenzyme was much greater than that of Pol γα, which is consistent with previous reports (Gray and Wong, 1992). mtSSB had no effect on the exonucleolytic activity of Pol γα in this case, whereas it was inhibitory to the activity of the holoenzyme.

### Loop 2.3 Weakens the Ability of mtSSB to Interact Directly With Pol γα and the DNA Template

We reported previously that a variant of mtSSB lacking nine residues of loop 2.3 (i.e., Δ67-75), here denoted as mtSSB_l2.3_, fails to stimulate processive DNA synthesis by Pol γ holoenzyme (Oliveira and Kaguni, 2011; Ciesielski et al., 2015; Figure 4A). Here we tested whether this also applies to the activity of the Pol γα subunit. We observed that, in contrast to Pol γ holoenzyme, mtSSB_l2.3_ stimulated the activity of Pol γα. Moreover, the stimulatory effect of mtSSB_l2.3_ was 3–4-fold greater than in the case of the wild-type protein (Figure 4A), approaching the nucleotide incorporation by Pol γ holoenzyme under these conditions (Supplementary Figure 4). This suggested to us that the processivity of Pol γα is enhanced in the presence of mtSSB_l2.3_. Therefore, we tested the extent of primer elongation by Pol γα on the M13 ssDNA template, in the presence or absence of the mtSSB variant. We found no difference in the length of products generated by Pol γα, although shorter species became more apparent when mtSSB_l2.3_ was present in the reaction (Figure 4B, bold arrow). This indicates that the elevated stimulatory effect of mtSSB_l2.3_ on the activity of Pol γα does not result from the enhanced processivity of the enzyme.

It was suggested previously that loop 2.3 facilitates a repulsive interaction of mtSSB with Pol γ, enabling the displacement of the former from the template (Cerrón et al., 2019). We tested whether the lack of loop 2.3 affects the Pol γα-mtSSB interaction by biolayer interferometry, using sensors saturated with BTN-mtSSB_l2.3_. We found that Pol γα binds to mtSSB_l2.3_ with a K_D_=26nM (±4.9nM; Figure 4C), which is approximately 2-fold stronger than in the case of the wild-type mtSSB. To test whether loop 2.3 is relevant for the displacement of mtSSB from the DNA template, we examined the efficiency of primer extension over a stretch of 44nt, in the presence or absence of wild-type versus loop 2.3-deficient mtSSB (Figure 4D). Consistent with the earlier results (Figures 3A,C), the wild-type mtSSB did not affect the activity of Pol γα when used at 1:1 molar ratio to DNA template, but reduced its activity slightly (~10%) only when used at 3-fold excess. In contrast, mtSSB_l2.3_ inhibited primer extension by ~10% at 1:1 molar ratio to DNA template, and by ~25%, at the three-fold excess. These results indicate that while Pol γα can displace the wild-type mtSSB to some extent, the lack of loop 2.3 impedes this activity and consequently, limits DNA synthesis.

The reduced capacity of Pol γ to displace mtSSB_l2.3_ may result from an elevated DNA-binding affinity of the latter, which we suggested previously (Oliveira and Kaguni, 2011). To evaluate this, we compared the ssDNA-binding affinity of the wild-type and loop2.3-deficient mtSSB by biolayer interferometry, using sensors saturated with a 40nt-long ssDNA oligomer. In both cases, the binding traces indicated a non-1:1 binding, likely due to binding cooperativity and multivalency of both the ligand and the analyte (Supplementary Figure 5). In addition, mass transfer interference was evident. Because of these limitations we chose to estimate DNA-binding affinities from the equilibrium responses of best fitted curves. We found a K_D_ of 2.3nM for the wild-type mtSSB, and 1.4nM for mtSSB_l2.3_ (Figure 4E). This indicates that the lack of loop 2.3 enhances ssDNA binding by mtSSB. Notably, these values, as well as the resulting difference between them, are in very good agreement with our earlier estimations by gel mobility shift assay of 3.8nM for wild-type mtSSB, and 2.4nM for mtSSB_l2.3_ (Oliveira and Kaguni, 2011).

Finally, we assessed the effect of the loop 2.3-deficient mtSSB on the exonucleolytic activity of Pol γα (Figure 3D). We observed that in the absence of mtSSB binding sites (5nt gap), mtSSB_l2.3_ stimulated primer excision by Pol γα to the same extent as the wild-type protein, whereas it had no effect on the activity of the holoenzyme. This agrees with our interpretation that the attraction between the proteins contributes to loading of Pol γα onto short gaps. When mtSSB_l2.3_ was able to bind to the DNA substrate (primer-template), the exonucleolytic processivity of Pol γα was reduced to approximately a single nucleotide, while the overall abundance of the products remained similar to that generated by the wild-type mtSSB, or Pol γα alone. This suggests that in the absence of the repulsive loop 2.3, mtSSB halts the advancement of Pol γα when bound to a downstream stretch of ssDNA, increases its dissociation and consequently, substrate turnover. In the case of the holoenzyme, the effect of mtSSB_l2.3_ was inhibitory to both the efficiency and processivity of exonucleolysis, as in the case of the wild-type mtSSB.

## Discussion

In this study, we aimed to evaluate the physical interaction between mtSSB and Pol γ, and demonstrated for the first time that mtSSB interacts physically with Pol γα. The fact that this interaction occurs in the absence of a C-terminal domain, which in cases of both the homologous SSB protein of *E. coli* or non-homologous SSBs of phages T7 and T4, is necessary to bind cognate DNA polymerases (Oliveira and Ciesielski, 2021), makes our finding unique. Notably, the interaction occurs only in the absence of the accessory subunit Pol γβ. In agreement with this observation, we reported previously that under similar experimental conditions, the binding affinity between the two Pol γ subunits (K_D_ 20nM) is over two-fold stronger than that which we report here for the Pol γα-mtSSB interaction (Ciesielski et al., 2016a). In the other replication systems, binding of the accessory subunit enhances the interaction of polymerases with SSB proteins, which in turn increases the efficiency of DNA synthesis (Kelman et al., 1998; Salinas and Benkovic, 2000; Hernandez and Richardson, 2019). This contrasts with the case of the mitochondrial counterparts, as we observed that the affinity for mtSSB binding is inversely related with the efficiency of DNA synthesis, especially at high mtSSB concentrations (Figure 3D; Supplementary Figure 4). These differences imply that polymerase- SSB interactions in different biological systems may have distinct physiological roles.

Pursuing the functional relevance of the mtSSB-Pol γα interaction, we found that mtSSB facilitates loading of Pol γα onto short gap sites, thereby increasing the efficiency of primer excision (Figure 3D). This, together with the exclusive ability of Pol γα (i.e., but not holoenzyme) to excise 8-oxoguanine (Wong et al., 2009), implies that the interaction with mtSSB may facilitate the engagement of Pol γα in excision-based mtDNA repair mechanisms. Such mechanisms involve a gap-filling step, and although we found that mtSSB had no effect on this activity of Pol γα, the higher fidelity of Pol γα, together with its inability in strand displacement (Figure 3C), argue that it may serve in this capacity as well. Conversely, in addition to its inability to excise short gaps, Pol γ holoenzyme exhibits lower fidelity, and relatively-effective strand displacement synthesis, which generates long non-ligatable 5'-flaps that are deleterious to mtDNA replication (Macao et al., 2015). Notably, the *in vivo* expression levels of the proteins under study (Figure 2E) suggest that Pol γα is present in human tissues at a level sufficient to perform the putative repair function, in addition to its role as the component of the replisome. Interestingly, the fact that primer excision was stimulated by mtSSB only in the absence of a sufficiently-long binding site for it on the DNA substrate might suggest that the putative repair occurs post-replication, when mtSSB binding sites would likely be limited, thus resulting in a pool of mtSSB available to load Pol γα onto short gaps. On the other hand, binding of mtSSB to the primer-template did not stimulate the exonucleolytic activity of Pol γα and inhibited that of the holoenzyme, which, taken together with the fact that binding of mtSSB to ssDNA template stimulates processive DNA synthesis (Figure 3A), suggests that the presence of mtSSB on the DNA template favors mtDNA replication over repair/excision functions, which has potential implications for mitochondrial/cellular regulation.

We proposed earlier that negatively-charged loop 2.3 moieties extrude from the surface of the mtSSB tetramer to face the advancing Pol γ (Figure 1), and mediate a repulsive interaction that drives the unwrapping and displacement of mtSSB from the DNA template (Cerrón et al., 2019). This putative mechanism is supported by the two-fold increase in the Pol γα-mtSSB binding affinity upon loop 2.3 deletion. The proposed mechanism also assumes that a lack of loop 2.3 diminishes the repulsive character of the interaction, thereby decreasing the efficiency of mtSSB displacement, which also agrees with our observations (Figure 4D). The relevance of the repulsive interaction between mtSSB and Pol γ for mtDNA synthesis is also corroborated by the inhibitory effect of high salt concentrations on Pol γ activity that we demonstrated previously (Oliveira and Kaguni, 2011). High ionic strength is likely to eliminate the electrostatic repulsion between the two proteins, and in turn disable the ability of Pol γ to displace mtSSB. We also observed that the presence of loop 2.3 weakens the DNA binding activity of mtSSB, as in its absence the affinity of mtSSB for ssDNA increases ~2-fold (Figure 4E). This effect can again be attributed to the negative charge of the loop, which may be repulsive to the negatively-charged DNA strand. Tighter binding to DNA by mtSSB_l2.3_ may contribute to the reduced ability of Pol γα to displace it (Figures 4B,D).

The reduced capability to displace mtSSB upon deletion of loop 2.3 may be the direct cause of the elevated efficiency of Pol γα activity in the processive DNA synthesis assay (Figure 4A). We observed that the stimulatory effect of mtSSB occurs only when the DNA template contains long stretches of ssDNA that may allow non-productive binding of Pol γα (Figures 3A,B). In contrast, we observed no stimulation of Pol γα activity in assays using DNA substrates that allow only productive binding of Pol γα (Figures 3C, 4D). Taken together, these observations argue that mtSSB increases the activity of Pol γα by restricting its non-productive residence on unprimed ssDNA stretches, thereby increasing the frequency of the primer binding. Deletion of loop 2.3 may enhance this mechanism, as mtSSB_l2.3_ limits the advancement of Pol γα more efficiently than the wild-type protein (Figures 4B,D), which in turn could promote DNA substrate turnover. This is supported directly by our finding that the processivity of the exonucleolytic activity of Pol γα in the presence of downstream-bound mtSSB_l2.3_ is reduced to a single nucleotide, while the overall abundance of exonucleolytic products remains unchanged (Figure 3D).

## Data Availability Statement

The original contributions presented in the study are included in the article/Supplementary Material; further inquiries can be directed to the corresponding authors.

## Author Contributions

LK and GC designed the experimental approach and analyzed the data. GC executed the biolayer interferometry experiments and processivity assays, and drafted the manuscript. SK and CB conducted gel-based assays. LK provided critical comments and edited the manuscript. All authors contributed to the article and approved the submitted version.

## Conflict of Interest

The authors declare that the research was conducted in the absence of any commercial or financial relationships that could be construed as a potential conflict of interest.

## Publisher’s Note

All claims expressed in this article are solely those of the authors and do not necessarily represent those of their affiliated organizations, or those of the publisher, the editors and the reviewers. Any product that may be evaluated in this article, or claim that may be made by its manufacturer, is not guaranteed or endorsed by the publisher.

## Footnotes

^1^ http://www.proteinatlas.org
