# Supplementary material for: Physical and Functional Interaction of Mitochondrial Single-Stranded DNA-Binding Protein and the Catalytic Subunit of DNA Polymerase Gamma
Source: Front Genet. 2021 Sep 1;12:721864. doi: 10.3389/fgene.2021.721864 (PMC8440931; doi:10.3389/fgene.2021.721864)
Supplement: Supplementary file 1 [file Data_Sheet_1.pdf]

Physical and functional interaction of mitochondrial single-stranded DNA-binding protein and the catalytic subunit of DNA polymerase gamma

Grzegorz L. Ciesielski[1,2,3*], Shalom Kim[3], Carolina de Bovi Pontes[3], Laurie S. Kaguni[1,2*]

[1] Department of Biochemistry and Molecular Biology and Center for Mitochondrial Science and Medicine, Michigan State University, East Lansing, MI 48824, USA,

[2] Institute of Biosciences and Medical Technology, University of Tampere, FI-33014 Tampere, Finland,

[3] Department of Chemistry, Auburn University at Montgomery, Montgomery, AL 36117, USA

Supplementary Materials

Nucleotides, nucleic acids and proteins

Unlabeled deoxyribonucleotides were purchased from Qiagen. [α-^{32}P]dCTP was purchased from PerkinElmer Life Sciences. Cy3-dUTP was purchased from PromoCell. Single-primed bacteriophage M13 ssDNA (6,407 nt) was as described previously (Williams et al., 1993). DNase I-activated calf-thymus DNA was purchased from Sigma and prepared as described previously (Wernette et al., 1988). The following modified or unmodified DNA oligomers were purchased from Integrated DNA Technologies: for the ssDNA-mtSSB binding BLI experiments (Figure 4E) BTN-ssDNA 40-mer 5’-BTN-ATTATGAATTAATTTAATAATTTTTTTTTTTTTTTTTTTT-3′ was used; for the single gap and exonuclease assays fluorescently-labeled 15-mer primer 5'-Cy3-CGCCAGGGTTTTCCC-3' was combined with 40-mer 5’-GATGCTACCTGAAGTGATTGTGACTGGGAAAACCCTGGCG-3’ or 70-mer 5’-GATGCTACCTGAAGTGATTGATTGATTACGATGAACATGAACTCGATGCTAGACTGGGAAAACCCTGGCG-3’ template strands, and 20-mer 5’-CAATCACTTCAGGTAGCATC-3’ or 60-mer 5’-TTTTTTTTTTTTTTTTTTTTTTTTTTTTTTTTTTTTTTTTCAATCACTTCAGGTAGCATC-3’ complimentary strand, to generate substrates depicted in Figures 3C, D; for the mtSSB displacement and exonuclease assays the same as above fluorescently-labeled 15-mer primer was used in combination with unlabeled 59-mer template: 5'-ATTAGAATGAACATGAATTCGATGCTACCTGAAGTGATTGGACTGGGAAAACCCTGGCG-3'. Primer-template and the single gap DNA substrates were prepared by heating up an equimolar mixture of oligomers in 10 mM Tris-HCl pH 8.0, 50 mM NaCl for 10 minutes at 95 °C and allowing the mixture to cool down overnight. Pol γα, Pol γβ, mtSSB and $mtSSB_{I2.3}$ were purified as described previously (Ciesielski et al., 2015). Biotinylation of mtSSB and $mtSSB_{I2.3}$

was carried out using Thermo Scientific EZ-Link NHS-Biotin kit (Thermo Scientific) according to manufacturer's guidelines (see also Supplementary Figure 1 for details).

Supplementary Figures

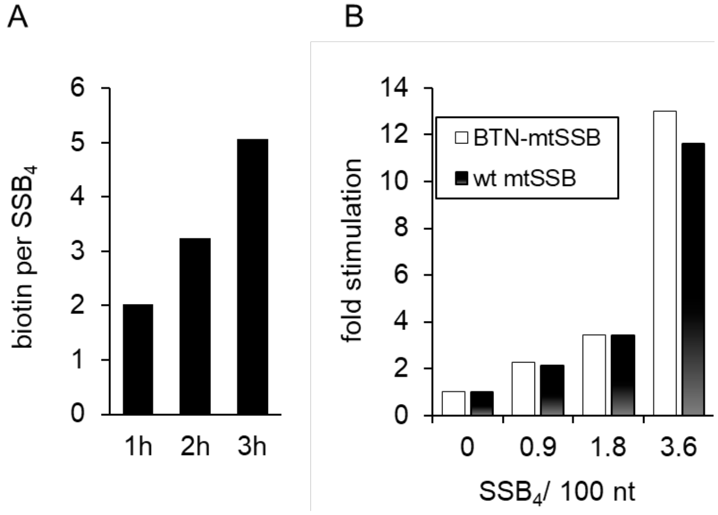

Supplementary Figure 1. Biotinylation of mtSSB does not alter its ability to stimulate the activity of Pol γ. A) Biotinylation of mtSSB was carried out using Thermo Scientific EZ-Link NHS-Biotin kit (Thermo Scientific) according to the manufacturer's guidelines. Briefly, mtSSB was dialyzed against gf buffer 50 mM KPi pH = 7.6, 100 mM NaCl, 2mM EDTA, 2 mM DTT, 8% glycerol, and combined with NHS-Biotin at 1:40 molar ratio. Reactions was carried out for 1, 2, or 3 hours on ice and biotinylated (BTN-)mtSSB was purified by gel filtration on the Superdex 200 column (General Electric). The conjugation of mtSSB with biotin was confirmed by streptavidin-blot. The number of biotins conjugated to a single mtSSB tetramer was estimated using the HABA colorimetric assay (Thermo Scientific), according to the manufacturer's instructions, and the results are presented in the graph. The preparation of BTN-mtSSB obtained after 2 hours reaction was used for all other analyses presented in this work. B) The effect of biotinylation on the ability of mtSSB to stimulate Pol γ activity was assessed by the processive DNA synthesis assay using mtSSB (light bars) or BTN-mtSSB (dark bars) at the indicated molar ratio to available binding sites on the DNA substrate (i.e. SSB_4/ 100 nt), as described under Materials and Methods.

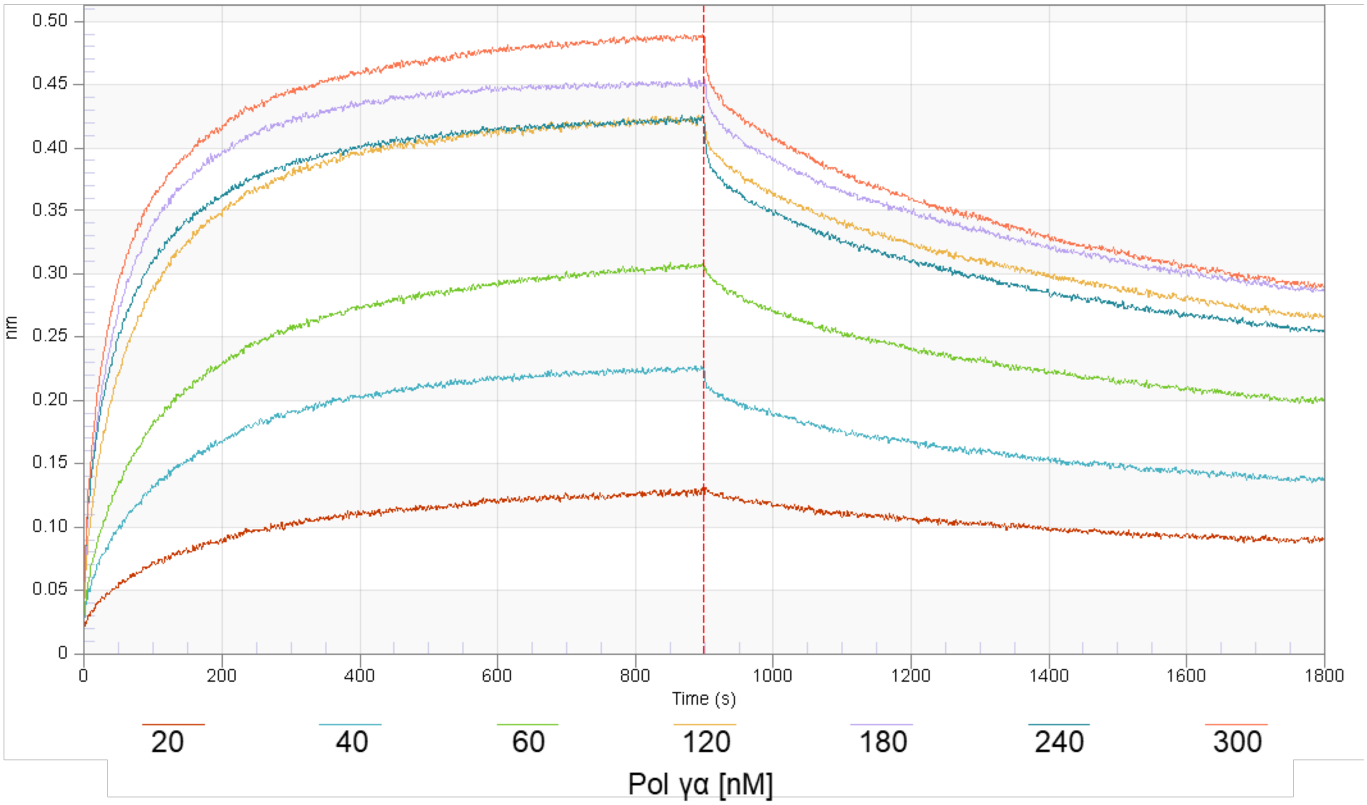

Supplementary Figure 2. Representative sensogram of Pol γα binding to mtSSB-coated sensors. The BLI experiment was performed as described under Materials and Methods and Figure 2A, using indicated Pol γα concentrations. The sensogram represents a set of results corresponding to Figure 2B.

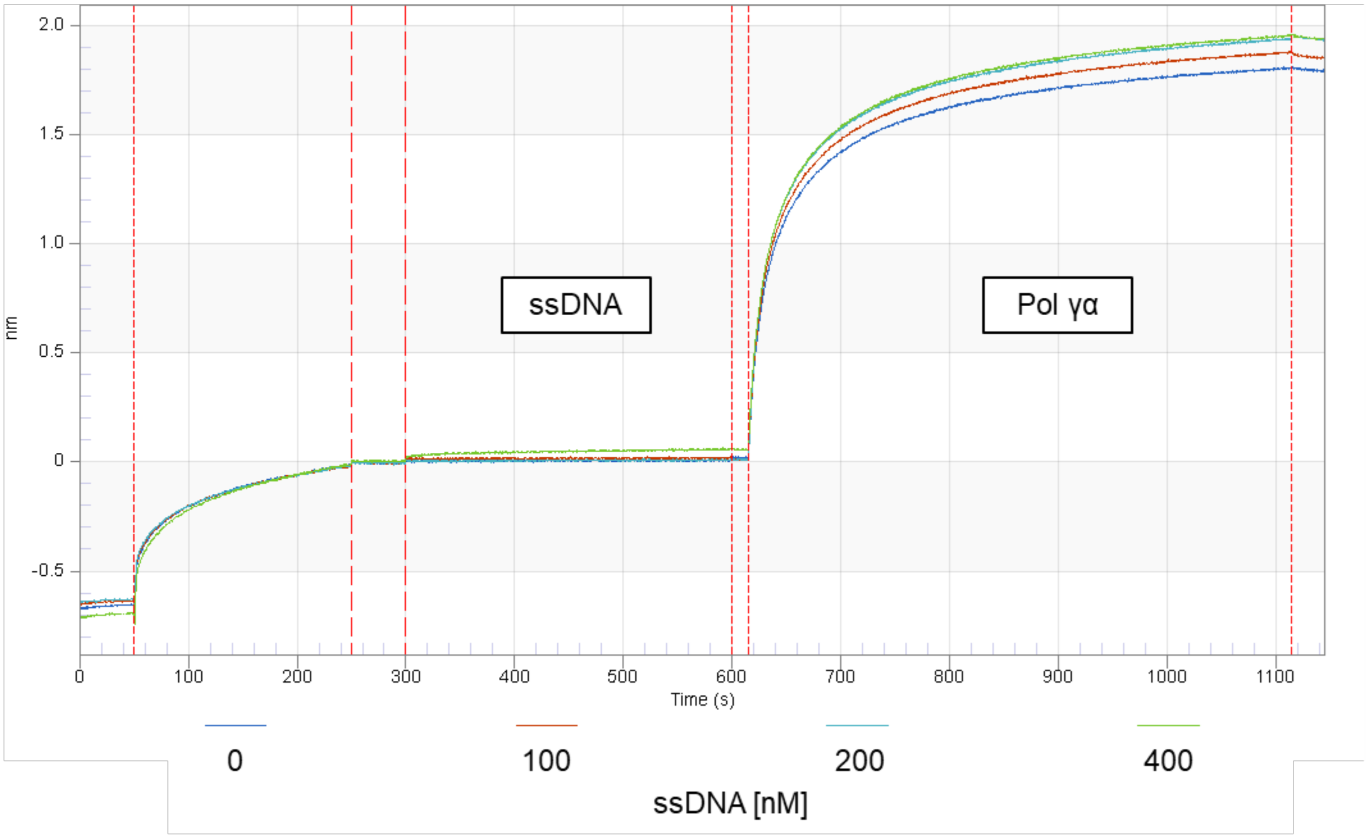

Supplementary Figure 3. Pre-binding of ssDNA to mtSSB-coated sensor does not interfere with Pol γα binding. The BLI experiment was performed as described under Materials and Methods, except that after blocking with BSA, mtSSB-sensors were placed into solutions of increasing concentration of 43-nts DNA oligomer as indicated, followed by brief washing and association of 120 nM solution of Pol γα.

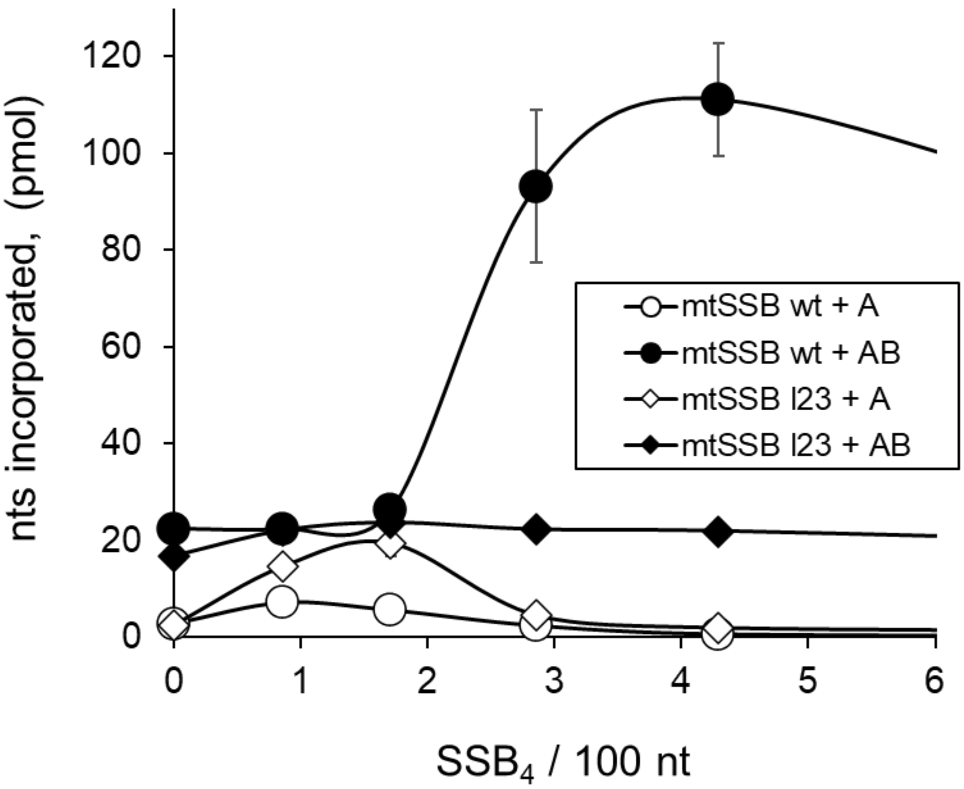

Supplementary Figure 4. The graph represents the results of the processive DNA synthesis assays presented in Figures 3A and 4A, as pmol of nucleotide incorporated, instead of the fold of stimulation.

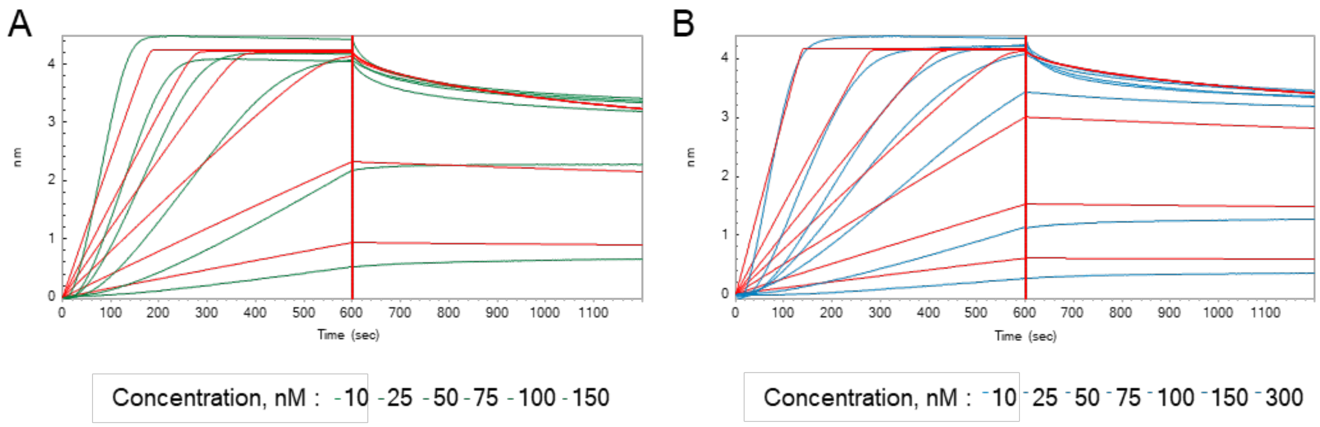

Supplementary Figure 5. The diagrams indicate the results of biolayer interferometry analysis of the ssDNA-binding affinity of the wild-type (A) and mtSSB$_{12.3}$ (B) variants, described under Materials and Methods. Red curves indicate mass transfer fits, which were used to generate the graph represented in Figure 4E.
